# Altered Protein Expression of Cardiac CYP2J and Hepatic CYP2C, CYP4A, and CYP4F in a Mouse Model of Type II Diabetes—A Link in the Onset and Development of Cardiovascular Disease?

**DOI:** 10.3390/pharmaceutics9040044

**Published:** 2017-10-12

**Authors:** Benoit Drolet, Sylvie Pilote, Carolanne Gélinas, Alida-Douce Kamaliza, Audrey Blais-Boilard, Jessica Virgili, Dany Patoine, Chantale Simard

**Affiliations:** 1 Faculté de Pharmacie, Université Laval, 1050 Avenue de la Médecine, Québec, QC G1V 0A6, Canada; carolanne.gelinas.1@ulaval.ca (C.G.); lovedk90@hotmail.com (A.-D.K.); audrey.blais-boilard.1@ulaval.ca (A.B.-B.); jessica.virgili.1@ulaval.ca (J.V.); chantale.simard@pha.ulaval.ca (C.S.); 2 Centre de Recherche, Institut Universitaire de Cardiologie et de Pneumologie de Québec, 2725 Chemin Sainte-Foy, Québec, QC G1V 4G5, Canada; sylvie.pilote@criucpq.ulaval.ca (S.P.); dany.patoine@criucpq.ulaval.ca (D.P.)

**Keywords:** arachidonic acid, CYP450 enzymes, Type II diabetes, cardiovascular disease

## Abstract

Arachidonic acid can be metabolized by cytochrome P450 (CYP450) enzymes in a tissue- and cell-specific manner to generate vasoactive products such as epoxyeicosatrienoic acids (EETs-cardioprotective) and hydroxyeicosatetraenoic acids (HETEs-cardiotoxic). Type II diabetes is a well-recognized risk factor for developing cardiovascular disease. A mouse model of Type II diabetes (C57BLKS/J-*db/db*) was used. After sacrifice, livers and hearts were collected, washed, and snap frozen. Total proteins were extracted. Western blots were performed to assess cardiac CYP2J and hepatic CYP2C, CYP4A, and CYP4F protein expression, respectively. Significant decreases in relative protein expression of cardiac CYP2J and hepatic CYP2C were observed in Type II diabetes animals compared to controls (CYP2J: 0.80 ± 0.03 vs. 1.05 ± 0.06, *n* = 20, *p* < 0.001); (CYP2C: 1.56 ± 0.17 vs. 2.21 ± 0.19, *n* = 19, *p* < 0.01). In contrast, significant increases in relative protein expression of both hepatic CYP4A and CYP4F were noted in Type II diabetes mice compared to controls (CYP4A: 1.06 ± 0.09 vs. 0.18 ± 0.01, *n* = 19, *p* < 0.001); (CYP4F: 2.53 ± 0.22 vs. 1.10 ± 0.07, *n* = 19, *p* < 0.001). These alterations induced by Type II diabetes in the endogenous pathway (CYP450) of arachidonic acid metabolism may increase the risk for cardiovascular disease by disrupting the fine equilibrium between cardioprotective (CYP2J/CYP2C-generated) and cardiotoxic (CYP4A/CYP4F-generated) metabolites of arachidonic acid.

## 1. Introduction

Arachidonic acid (AA) is an essential polyunsaturated fatty acid notably found in cell membrane phospholipids. It has an important role in a number of cell signaling and regulation pathways upon its cleavage from membranes via a sequence of tightly regulated events [1]. The cleavage sequence is initiated by the activation of a calcium-dependent type IV phospholipase A2 in response to a stimulus, such as ischemia [2]. The released non-esterified AA acts as a substrate for oxidation by cyclooxygenases (COX), lipoxygenases (LOX), and cytochrome P450 (CYP450) enzymes in the heart and elsewhere, thereby generating a cascade of lipid second messengers, orchestrating a broad range of critical physiological processes, including hemodynamic functions [1,2,3]. Both the lipoxygenase [4] and the cyclooxygenase [5] pathways have been thoroughly studied and described. Briefly, via the LOX pathway, AA is converted into a large number of lipid mediators with vasoactive and immunomodulatory effects such as lipoxins, leukotrienes, hepoxilins, eoxins, resolvins, protectins, and others [4]. On the other hand, cyclooxygenase (COX), with both its COX-1 and COX-2 isoforms, transforms AA in either prostaglandins, prostacyclins, or thromboxane; other products are known for their vasoactive, immunomodulatory, and platelet-aggregating effects [5]. In contrast, the CYP450 pathway, also known as the third pathway, was far less understood [1,6]. However, this pathway is increasingly recognized as a ‘yin and yang’ in relation to its potential opposing effects on the cardiac function, depending on which subfamily of the CYP enzyme is metabolizing AA. Indeed, when it is transformed by epoxidation, AA generates a number of products known as epoxyeicosatrienoic acids (EETs) that promote vasodilation, angiogenesis, and thrombolysis. It then also inhibits inflammation, smooth muscle cell migration, and apoptosis, which together lead to a preserved cardiac function [7]. In contrast, when AA is hydroxylated, it produces compounds such as hydroxyeicosatetraenoic acids (HETEs) that promote vasoconstriction, inflammation, smooth muscle cell migration, and apoptosis, all leading to cardiac dysfunction [7]. In fact, CYP450 enzymes can metabolize AA in a tissue- and cell-specific manner to generate major and highly relevant derivatives, such as cardioprotective EETs and cardiotoxic HETEs. These epoxygenated and hydroxylated lipid derivatives are known to have various biological functions, such as regulating the vascular tone and reactivity, renal and pulmonary functions, ion transport, and growth response [8]. The epoxygenases (CYP2C and CYP2J) generate EETs, while the ω-hydroxylases (CYP4A and CYP4F) generate HETEs [2,6,9]. The human CYP2C subfamily consists of four members (CYP2C8, CYP2C9, CYP2C18, and CYP2C19) and they account for approximately 20% of the P450 enzymes in the human liver. Moreover, they are expressed to variable extents in a number of tissues such as liver, kidney, gut, brain, lungs, aorta, and importantly, in the heart [10,11]. The human CYP2J subfamily has only a single gene, CYP2J2 metabolizing AA [12]. CYP2J2 was detected in tissues such as lung, intestine, liver, pancreas, and seminal vesicles, and is particularly abundant in the cardiovascular tissue [13,14,15,16,17]. Enzymes of the CYP4A subfamily have been identified in virtually all mammalian species. In human, CYP4A11 and CYP4A12 have been identified. The CYP4A fatty acid ω-hydroxylases are ubiquitously expressed and consistently found in strong levels in the kidney, liver, lung, intestine, skeletal muscle, and heart [18]. The mammalian CYP4F subfamily consists of seven human forms: CYP4F2 (liver and kidney), CYP4F3A (bone marrow and neutrophiles), CYP4F3B (liver and kidney), CYP4F8 (prostate), CYP4F11 (liver, kidney, heart, and skeletal muscle), CYP4F12 (gastrointestinal tract, heart, and kidney), and CYP4F22 (skin). Therefore, both CYP2 and CYP4 families are present in the heart.

CYP-derived HETEs have deleterious effects in the heart during ischemia. These include a significant pro-inflammatory effect during reperfusion and potent vasoconstriction in the coronary arteries [2]. In contrast, epoxidation of AA generates 5,6-, 8,9-, 11,12-, and 14,15-EET that have been shown to limit ischemia-reperfusion injury, to have potent anti-inflammatory effects within the vasculature, and to be potent vasodilators of the coronary arteries [2]. Once formed, EETs undergo hydrolysis by soluble epoxide hydrolase (sEH) to less biologically active dihydroeicosatrienoic acids (DHETs) [2]. Soluble epoxide hydrolase (sEH) is widely distributed in mammalian tissues. Indeed, it is present in vascular endothelia [19] and in vascular smooth muscle cells [20]. Dysregulation of lipid metabolism has frequently been associated with disorders such as inflammation, neoplasia, diabetes, and neurodegeneration, as well as cardiovascular disease [8]. In line with this, a probable link between CYP450 expression/activity and cardiovascular disease (CVD), such as high blood pressure, coronary artery disease, myocardial ischemia and infarction, congestive heart failure, stroke, dilated cardiomyopathies, and rhythm disturbances has been established [21]. It is therefore suggested that alterations in the expression and/or activity of specific CYP450 epoxygenase/hydroxylase and sEH enzymes are likely to affect the delicate balance between the cardiovascular effects of EETs, HETEs, and DHETs [2]. As a consequence, abnormalities in these pathways may contribute to and/or promote the pathogenesis of CVD [22].

Well-known to be a major contributor to the onset and development of CVD [23], Type II diabetes (T2D) is therefore thought to perturbate the fine equilibrium between cardioprotective and cardiotoxic CYP-generated metabolites of AA by contributing to the cardiotoxic side. Indeed, this modulation of AA metabolism is suggested to be associated with the onset of coronary vasoconstriction leading to endothelial dysfunction, atherosclerosis, and CVD in general [24]. Of note, while both COX- and LOX-associated cardiovascular alterations have been demonstrated in the *db/db* mouse model of T2 [25,26], such demonstration is far less clear when considering the CYP pathway.

It was therefore our objective to evaluate the T2D-induced disturbances in the CYP450 pathway of AA metabolism that may contribute to the observed increase in the risk of CVD.

## 2. Materials and Methods

### 2.1. Animal Model

Male 7-weeks old BKS.Cg-m +/+ Lepr^db^/J mice (*db/db*: T2D) and control C57BLKS/J wild-type mice were purchased from The Jackson Laboratory (Bar Harbor, ME, USA). The animals were fed with standard chow and water ad libitum. At 12 weeks-old, blood drawns were performed by using the submandibular vein, and, thereafter, the animals were killed by cervical dislocation. Livers and hearts were quickly harvested, washed with ice-cold PBS, snap-frozen in liquid nitrogen, and further kept at −80 °C. These experiments were carried out in accordance with the Guide to the Care and Use of Experimental Animals of the Canadian Council on Animal Care. The research protocol was approved by the Comité de protection des animaux de l’Université Laval (CPAUL; Authorization # 2009140-3, on 20 December 2011).

### 2.2. Western Blots of Liver Proteins

Western blotting experiments were performed as previously described [27] to assess CYP2C, CYP4A, and CYP4F protein expression. Briefly, samples of frozen liver were homogenized in an ice-cold lysis buffer (in mmol/L): Tris-HCl (pH 7.4) 10, sucrose 320, EDTA 1, dithiothreitol 1, and a protease inhibitor cocktail 0.1% from Sigma-Aldrich (St. Louis, MO, USA). Total protein content was evaluated with the DC protein assay kit (Bio-Rad, Mississauga, ON, Canada). Western blot analysis of 10 µg (CYP2C), 12 µg (CYP4A), and 20 µg (CYP4F) of proteins were used to evaluate the levels of these three CYPs in the livers. The Mini-Protean TGX Stain-Free Precast Gel (Bio-Rad) or standard SDS-PAGE gel was used. The membrane was incubated overnight at 4 °C with either anti-CYP2C9 (Ab48558), anti-CYP4A (Ab3573), or anti-CYP4F12 (Ab71565) polyclonal primary antibodies (all three 1:1000; Abcam, Toronto, ON, Canada). The membrane was washed and then incubated with a goat anti-rabbit IgG HRP-linked secondary antibody (SC2004; 1:1000; Santa Cruz Biotechnology, Mississauga, ON, Canada) conjugated with horseradish peroxidase. In the specific case of CYP4F proteins, the membrane was washed, stripped in SDS/β-mercaptoethanol solution at 55 °C for 15 min to enhance the signal strength [28], and then incubated with the secondary antibody conjugated with horseradish peroxidase. Chemiluminescent immunodetection was performed by using the Luminata™ Crescendo Western HRP Substrate (EMD Millipore, Billerica, MA, USA) and the ChemiDoc^TM^ imaging system from Bio-Rad. Expression of the proteins of interest was normalized against total lane density of loaded proteins based on Amido black staining (CYP4F) or stain-free gel technology (CYP4A and CYP2C). The linear dynamic range of sample was evaluated for each antibody to make sure that an appropriate sample loading was used.

### 2.3. Western Blots of Cardiac Protein

Samples of frozen ventricles were homogenized in an ice-cold lysis buffer (in mmol/L): Tris-HCl (pH 7.4) 15, NaCl 150, sodium vanadate 0.2, EGTA 1, MgCl_2_ 1, β-mercaptoethanol 10, Triton 1%, sodium deoxycholate 0.5%, and a protease inhibitor cocktail 0.1% from Sigma-Aldrich. Total protein content was evaluated with the DC protein assay kit (Bio-Rad). Western blot analysis of 25 μg of proteins was used to assess the level of CYP2J protein in the ventricles. The membrane was incubated overnight at 4 °C with anti-CYP2J2 (Ab76176) polyclonal antibody (1:1000; Abcam). The membrane was washed and then incubated with a goat anti-rabbit IgG HRP-linked secondary antibody (SC2004; 1:1000; Santa Cruz Biotechnology) conjugated with horseradish peroxidase. Chemiluminescent immunodetection was performed using the Luminata™ Crescendo Western HRP Substrate (EMD Millipore) and the ChemiDoc^TM^ imaging system from Bio-Rad. Expression of the proteins of interest was normalized against total protein loaded based on Amido black staining of whole lanes. Again, the linear dynamic range of the method was evaluated.

### 2.4. Data Analysis

Student’s *t*-test followed by a Mann-Whitney rank sum test (when the test for normality failed) were used to evaluate the differences between groups (SigmaPlot 12.5, Jandel Scientific Software, San Rafael, CA, USA). All the results are expressed as mean ± SEM. Statistical significance was set at *p* < 0.05.

## 3. Results

Table 1 shows body weight and biochemical blood parameters of mice at sacrifice (week 12). Many clinical features of human Type II diabetes were shown to be present in the *db/db* mice (T2D) at sacrifice: severe obesity, hyperglycemia, hyperinsulinemia, hypertriglyceridemia, and hypercholesterolemia; thus confirming the validity of this T2D animal model.

**Figure 1:**
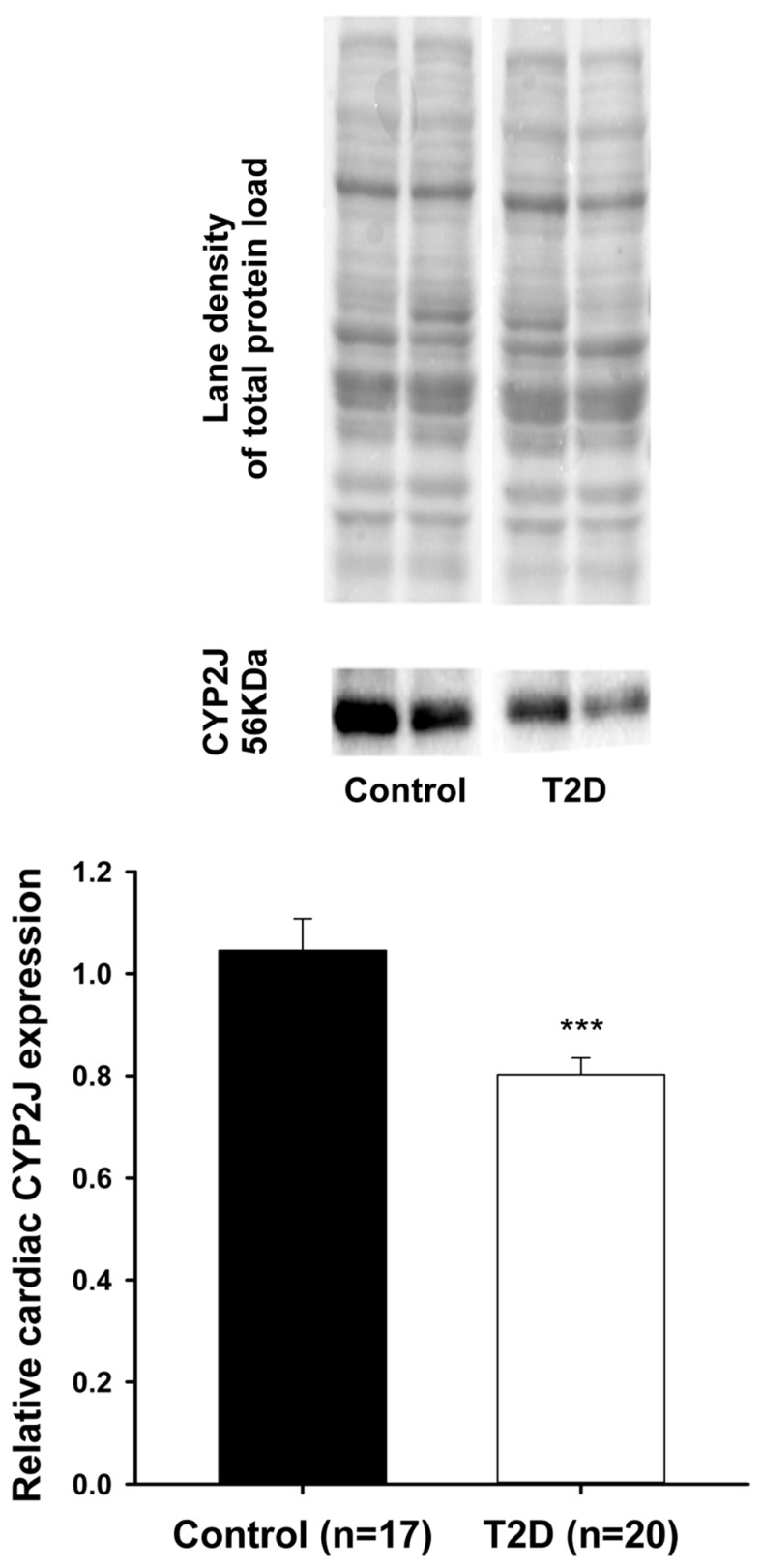
A significant decrease (*** *p* < 0.001) was observed in the relative protein expression of cardiac CYP2J in Type II diabetes (T2D) mice (0.80 ± 0.03) compared to controls (1.05 ± 0.06).

**Figure 2:**
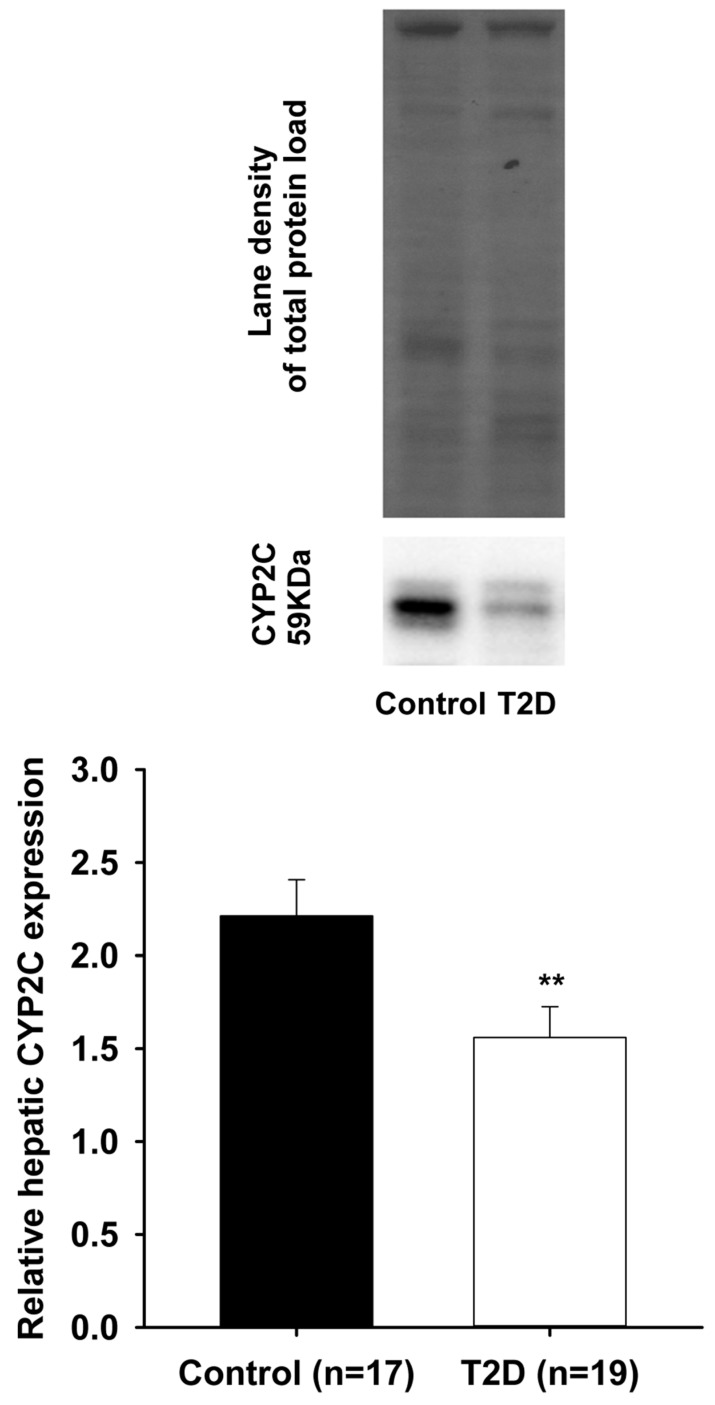
A significant decrease (** *p* < 0.01) was observed in the relative protein expression of hepatic CYP2C in T2D mice (1.56 ± 0.17) compared to controls (2.21 ± 0.19).

**Figure 3:**
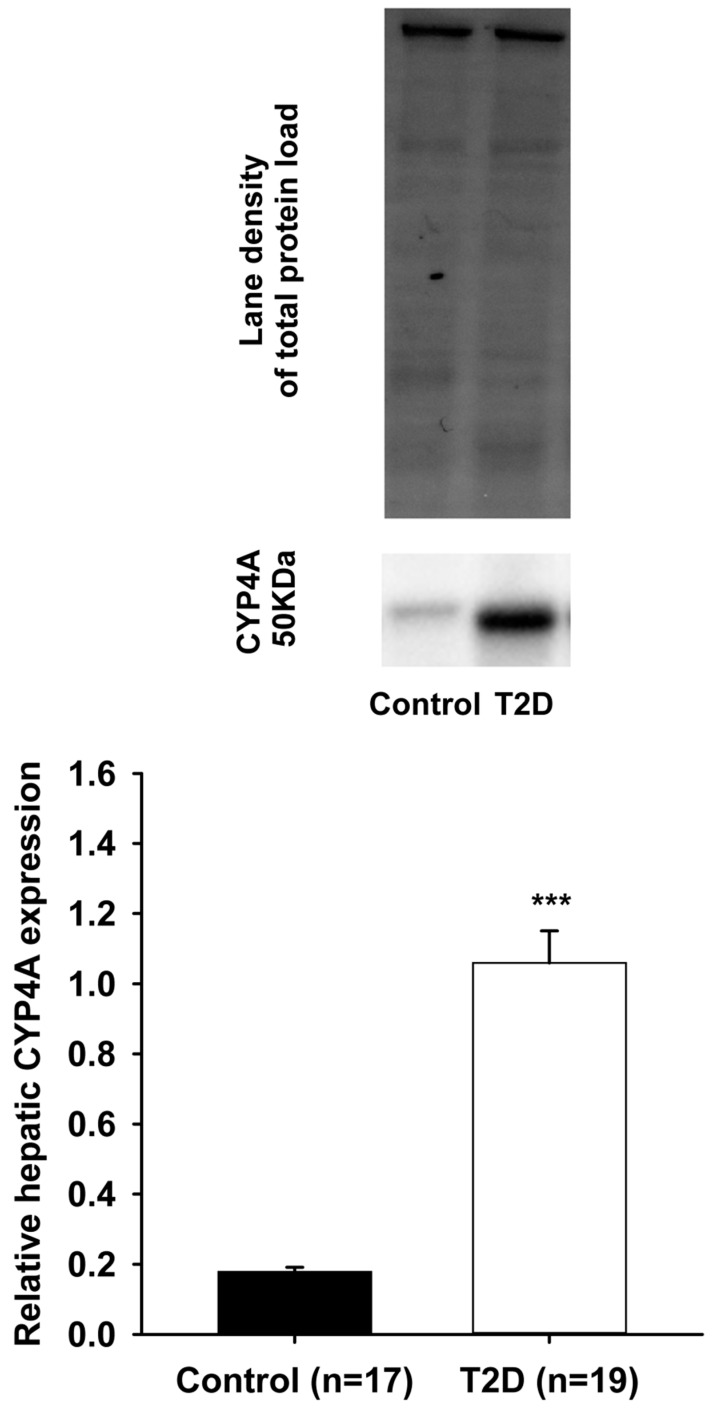
A significant increase (*** *p* < 0.001) was observed in the protein expression of hepatic CYP4A in T2D mice (1.06 ± 0.09) compared to controls (0.18 ± 0.01).

As shown in Figure 1 (lower panel), a significant reduction in relative expression of CYP2J proteins was observed in the cardiac ventricles of T2D mice, when compared to the control animals (0.80 ± 0.03 vs. 1.05 ± 0.06; *p* < 0.001). Upper panel shows the representative blot images of cardiac CYP2J and the total proteins loaded for both the control and T2D groups. Figure 2 (lower panel) also shows a significant reduction in relative expression of CYP2C proteins that was observed in the liver of T2D mice when compared to the control animals (1.56 ± 0.17 vs. 2.21 ± 0.19; *p* < 0.01). Upper panel shows the representative blot images of hepatic CYP2C and the total proteins loaded for both the control and T2D groups. In contrast, Figure 3 (lower panel) shows a significant increase in relative expression of CYP4A proteins that was observed in the liver of T2D mice when compared to the control animals (1.06 ± 0.09 vs. 0.18 ± 0.01; *p* < 0.001).

**Figure 4:**
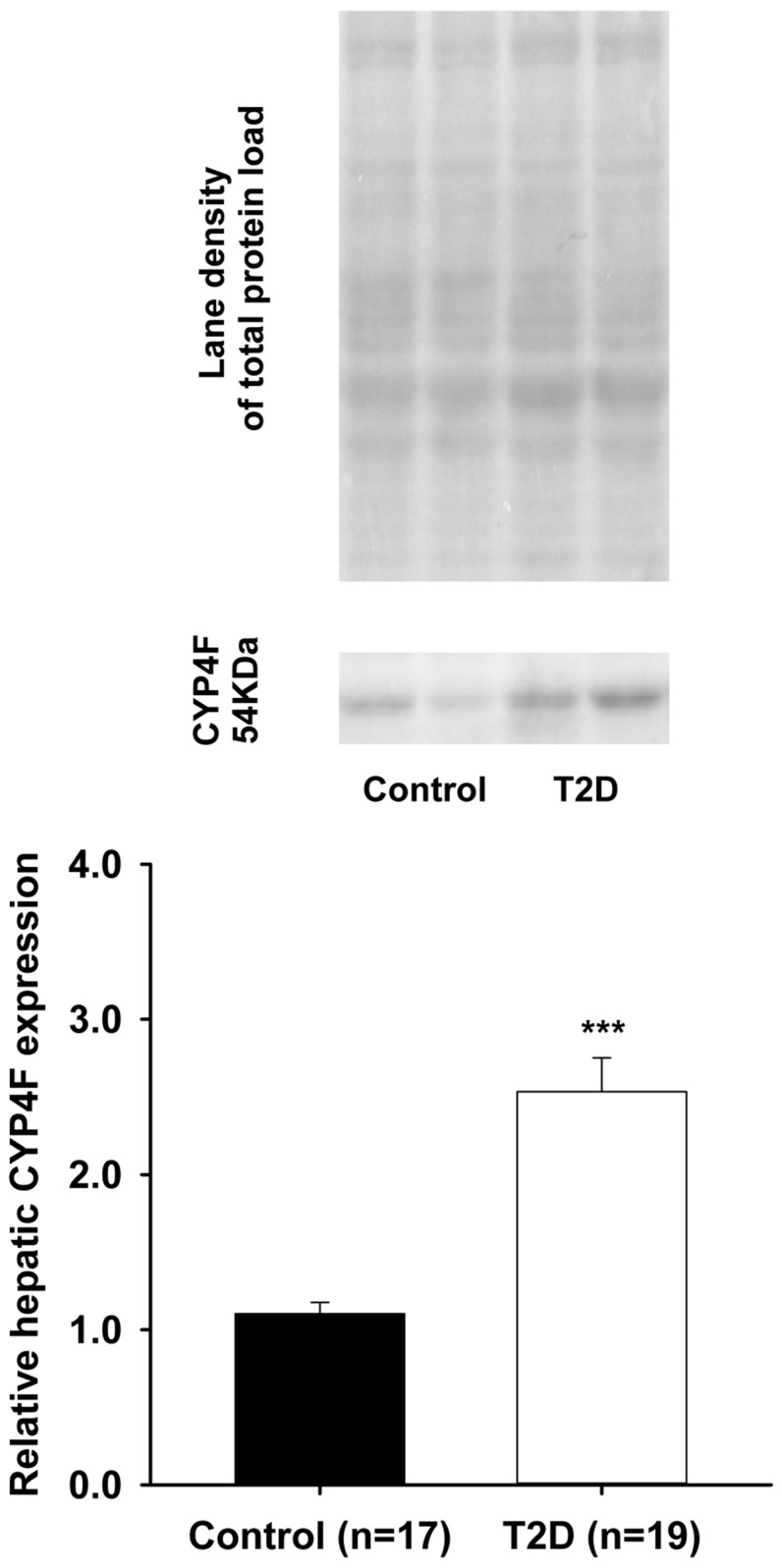
A significant increase (*** *p* < 0.001) was observed in the protein expression of hepatic CYP4F in T2D mice (2.53 ± 0.22) compared to controls (1.10 ± 0.07).

Upper panel shows the representative blot images of hepatic CYP4A and the total proteins loaded for both the control and T2D groups. Moreover, as seen with CYP4A, Figure 4 (lower panel) shows a significant increase in relative expression of CYP4F proteins that was observed in the liver of T2D mice when compared to the control animals (2.53 ± 0.22 vs. 1.10 ± 0.07; *p* < 0.001). Upper panel shows the representative blot images of hepatic CYP4F and the total proteins loaded for both the control and T2D groups.

## 4. Discussion

The pathophysiology of CVD is highly complex, and a constellation of risk factors contribute to its development and progression. Consequence of the current worldwide obesity epidemic, one major risk factor of CVD that is becoming epidemic as well is Type II diabetes. Interestingly, we have already shown that CYPs expression and metabolic activity are altered in a number of conditions such as metabolic syndrome and renal insufficiency in different animal models [29,30]. Besides, Imig et al. demonstrated a decrease in renal expression of CYP2C epoxygenase enzymes in diabetic obese Zucker rats and in high fat diet-fed insulin resistant rats [31]. They concluded that eicosanoids metabolites are altered in Type II diabetes and contribute to the progression of renal injury. Moreover, Shimojo et al. reported an induction of hepatic and renal CYP4A subfamily enzymes in diabetic rats [32]. Yousif et al. demonstrated that CYP4 family expression is twice as high in the hearts of diabetic rats when compared to normal animals [33]. Enriquez et al. showed that expression of murine CYP4A10 and 4A14 in the obese mice, and 4A2 in the male fatty Zucker rat, were greatly increased [34]. Zhao et al. [35] studied the mesenteric artery protein expression in lean and obese Zucker rats. They concluded that mesenteric arterial CYP2C11 and CYP2J proteins were decreased by 38% and 43%, respectively, in obese Zucker rats. In contrast, sEH mRNA and protein expressions were significantly increased in obese Zucker rat mesenteric arteries [35]. A previous study has shown that the synthesis of renal CYP450 (CYP)-derived eicosanoids is downregulated in genetic or high-fat diet-induced obese rats [36]. These results demonstrated that the PPAR-alpha agonist fenofibrate increased renal CYP-derived eicosanoids and restored endothelial dilator function in obese Zucker rats [36]. A study from Theken et al. [37] evaluated CYP epoxygenase (EET+DHET) and ω-hydroxylase (20-HETE) metabolic activity in liver and kidney in wild-type mice fed with a high fat diet, which promoted weight gain and significantly increased insulinemia. Hepatic CYP epoxygenase metabolic activity was significantly suppressed, while renal CYP ω-hydroxylase metabolic activity was significantly induced in high fat diet-fed mice. A significantly higher 20-HETE:EET+DHET formation rate ratio was observed in both tissues [37]. They concluded that the observed changes in CYP epoxygenase and hydroxylase metabolic activity were driven by high fat diet rather than by genotype.

Interestingly, there are recent clinical studies that begin to bridge the gap between animal models of CYP-mediated arachidonic acid metabolism and cardiovascular disease in humans. Indeed, Akasaka et al. [38] examined CYP2C19 genotypes in 81 patients with microvascular angina (MVA) caused by coronary microvascular dysfunction. They found that CYP2C19 poor metabolizers had declined levels of EETs, suggesting insufficient defensive mechanism against chronic inflammation, a risk factor for MVA. Moreover, Theken et al. [39] evaluated the role of CYP-derived eicosanoids in humans with stable atherosclerotic cardiovascular disease (CVD, *n* = 82) versus healthy volunteers (*n* = 36). Among other things, they noted that obesity was significantly associated with low plasma EET levels and 14,15-EET:14,15 DHET ratios. Collectively, their findings suggest that CYP-mediated eicosanoid metabolism is dysregulated in certain subsets of CVD patients and demonstrate that biomarkers of CYP epoxygenase are altered in stable CVD patients relative to healthy individuals. Later on, the same group came up with a study [24] of 106 patients with stable coronary artery disease (CAD) in which relationships between biomarkers of CYP-mediated eicosanoid metabolism and vascular function phenotypes were evaluated. Collectively, their findings demonstrated that enhanced CYP ω-hydroxylases (generating HETEs) and sEH (degradating EETs) metabolic functions are associated with more advanced endothelial dysfunction and vascular inflammation, respectively, in patients with established atherosclerotic CVD.

In the present study, we show that Type II diabetes, a major CVD-associated pathological condition, significantly decreases the cardiac protein expression of cytochrome P450 CYP2J and of hepatic CYP2C in *db/db* mice. As CYP2J and CYP2C are the major AA epoxygenases in the cardiovascular and hepatic systems [2], where they are widely expressed respectively [40], down regulation of these two CYP epoxygenases could compromise the formation of EETs, which play many crucial roles in cardiovascular homeostasis. In fact, in addition to their potent vasodilating effect, EETs have potent anti-inflammatory properties, inhibit platelet aggregation, promote fibrinolysis, and reduce vascular smooth muscle cell proliferation [41]. In addition, our results also show increased hepatic protein expression of both cytochrome P450 CYP4A and CYP4F in *db/db* mice. CYP4F2 is known to be the key AA ω-hydroxylase in human liver and kidney with a more superior substrate specificity for AA than the already established AA ω-hydroxylase CYP4A11 [42]. The massive formation of 20-HETE, along with its potent detrimental pro-inflammatory effects during ischemia-reperfusion and vasoconstrictor effect in the coronary arteries [2], suggests that it could also serve as an intracellular second messenger underlying the regulation of vascular tone [43].

## 5. Conclusions

Taken together, Type II diabetes-induced combined alterations of cardiac CYP2J, and hepatic CYP2C, CYP4A, and CYP4F protein expression are likely to play a significant role in CVD. As these four CYP isoforms were shown to generate the most vasoactive eicosanoid metabolites, Type II diabetes likely generates deleterious in situ alterations in the endogenous disposition of AA. Their net effect is thought to further promote CVD by disrupting the fine equilibrium between cardioprotective (CYP2C/CYP2J-generated) and cardiotoxic (CYP4A/CYP4F-generated) AA metabolites, adding weight on the cardiotoxic side of the balance.

## 6. Limitation

CVD-associated pathophysiological features of the diabetic mice such as (coronary disease, vasoconstriction, endothelial dysfunction, atherosclerosis, etc.) were not compared to those of the control animals. Such further studies, beyond the scope of this article, would help to elucidate how and how much the altered CYPs in Type II diabetes may influence the onset and progression of cardiovascular disease.

## Figures and Tables

**Table 1:** Body weight and biochemical blood parameters at sacrifice (week 12). (** *p* < 0.01, *** *p* < 0.001 vs. control).

Measures at Sacrifice (Week 12)	Control Mice (*n* = 17)	BKS.Cg-m +/+ Lepr^db^/J Mice (*db/db*: T2D) (*n* = 20)
Weight (g)	23.5 ± 0.5	41.3 ± 0.9 ***
Glycemia (mM)	8.2 ± 0.7	31.3 ± 0.6 ***
Insulinemia (ng/mL)	0.67 ± 0.06	3.90 ± 0.50 ***
HDL-C (mM)	2.10 ± 0.05	3.18 ± 0.13 ***
Triglyceridemia (mM)	1.67 ± 0.07	2.33 ± 0.22 **
Cholesterolemia (mM)	2.32 ± 0.17	3.77 ± 0.11 ***
